# Ex vivo lung perfusion: how we do it

**DOI:** 10.1007/s12055-021-01215-z

**Published:** 2021-09-01

**Authors:** John Santosh Murala, William Michael Whited, Amit Banga, Robert Castillo, Matthias Peltz, Lynn Custer Huffman, Amy Elizabeth Hackmann, Michael Erik Jessen, Fernando Torres, Michael Alton Wait

**Affiliations:** 1 Department of Cardiovascular and Thoracic Surgery, University of Texas Southwestern (UTSW) Medical Center, 5959 Harry Hines Blvd., 10th Floor, Suite HP10.110, Dallas, TX 75390 USA; 2 Division of Pulmonary and Critical Care Medicine, Department of Medicine, University of Texas Southwestern (UTSW) Medical Center, Dallas, TX USA; 3 Cardiovascular Intensive Care Unit and Previous EVLP Nursing Lead, University of Texas Southwestern Medical Center, Dallas, TX USA

**Keywords:** Lung transplantation, Ex vivo lung perfusion, Donor selection

## Abstract

Lung transplantation is an established treatment for patients with end-stage lung disease. However, a shortage of donors, low lung utilization among potential donors, and waitlist mortality continue to be challenges. In the last decade, ex vivo lung perfusion (EVLP) has expanded the donor pool by allowing prolonged evaluation of marginal donor lungs and allowing reparative therapies for lungs, which are otherwise considered not transplantable. In this review, we describe in detail our experience with EVLP including our workflow, setup, operative technique, and protocols. Our multidisciplinary EVLP program functions with the collaboration of surgeons, pulmonologists, and EVLP nurses who run the pump. EVLP program has been a valuable addition to our program. Since Food and Drug Administration (FDA) approval in 2019, we experienced incremental increased lung transplant volume of 12% annually.

## Introduction

Lung transplantation is an established treatment for patients with end-stage lung disease. However, there continues to be a shortage of available donor lungs. Nearly 80% of lungs are turned down from potential donors. Hence, the lung utilization for transplant is the lowest among all the solid organs, using 21% of lungs from brain-dead donors compared to 90% for kidney, 80% for liver, and 30–40% for heart [1]. The utilization rates among donation after circulatory death (DCD) donors are even lower at <2% of the lungs allocated for transplantation [2, 3]. According to recent Organ Procurement and Transplantation Network/Scientific Registry of Transplant Recipients (OPTN/SRTR) data for the year 2018, 2562 lung transplants (31% increase in 5 years) are performed in the United States (US) [4]. Unfortunately, even as this number has continued to increase, there is still an unmet need for transplantation as evidence by the waiting list of 3134 patients, which was an 8% increase over the previous year and a 42% increase over the past decade [4]. Sadly, 238 adult waitlisted patients died waiting for a suitable donor. This annual waitlist mortality rate of 7.6% has been constant over the years [4]. Increasing the donor pool remains of great interest to the transplant community; unfortunately, few donors meet the rather strict criteria of an ideal donor [5]. Recent years have witnessed the emergence of ex vivo lung perfusion (EVLP) as a method aimed at expanding the donor pool by repairing and reconditioning marginal donor lungs, not suitable for transplantation initially.

## Brief history

EVLP has been of interest within the scientific community for decades. In 1970, Jirsch et al. published a study where the authors developed an EVLP model with the hope of improving lung function in comparison to standard cold preservation [6]. In 2001, Steen et al. reported the first case of lungs evaluated on an EVLP circuit and transplanted from a DCD donor [7]. They subsequently performed animal studies on 12 pigs to demonstrate the proof of concept of recruiting lungs using EVLP from DCD donors [8]. In 2005, the same group performed the first human transplant of a non-acceptable donor lung, repaired using EVLP, after which the recipient had excellent lung function [9]. Steen continued to utilize EVLP and published a follow-up case series in 2009, where six transplants were performed utilizing rejected lung allografts, out of nine total evaluated [10]. The Toronto group, building on this work, published their landmark study in the *New England Journal of Medicine* in 2011 [11]. The authors placed 23 high-risk donor organs on EVLP and ultimately transplanted 20 organs with no significant difference in primary graft dysfunction at 72 h and no adverse events related to the utilization of EVLP.

Many groups in the literature have reported EVLP utilization [12–17].

In the US, a comprehensive trial called the NOVEL trial was undertaken to evaluate the safety of the EVLP. This was a multicenter prospective clinical trial utilizing EVLP to evaluate marginal donor lungs. During this trial, the utilization rate of lungs placed on EVLP was 55%, and the short-term outcomes, when compared to standard donors, were equivalent [18].

## EVLP program

Successful EVLP programs are very expensive and need large upfront investment with significant ongoing resource commitment towards supplies and personnel. Conventionally, the EVLP is housed in the operating room (OR) with lung transplant surgeons as the team leaders and the perfusion team managing the EVLP pump. This model is very familiar to surgeons with established service line responsibilities. However, this workflow can disrupt the existing clinical load, routine OR schedule, scheduling, and space issues, and potentially overload surgeons and clinical perfusionists.

### How we do it

**Fig. 1:**
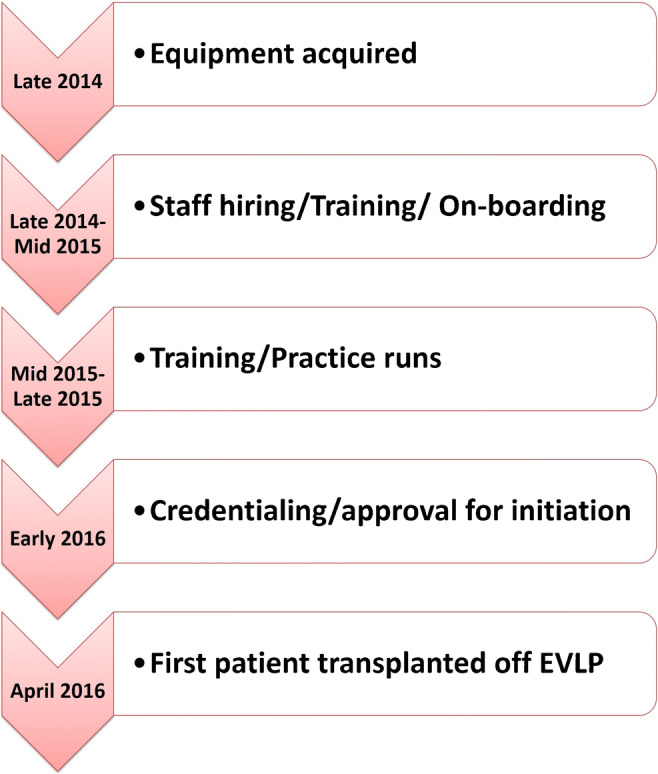
The time line for EVLP program at our institution (EVLP, ex vivo lung perfusion)

The shortage of quality donor lungs remains a major limiting feature for lung transplant programs. Despite performing close to 900 lung transplants at our institution, there remains a significant waitlist time for certain patients. The EVLP program was initiated at our center in 2014 as part of the multicenter NOVEL study [19]. Figure 1 shows the time line for our program. The setting up of the EVLP program also coincided with the initiation of the DCD lung program at our center.

**Fig. 2:**
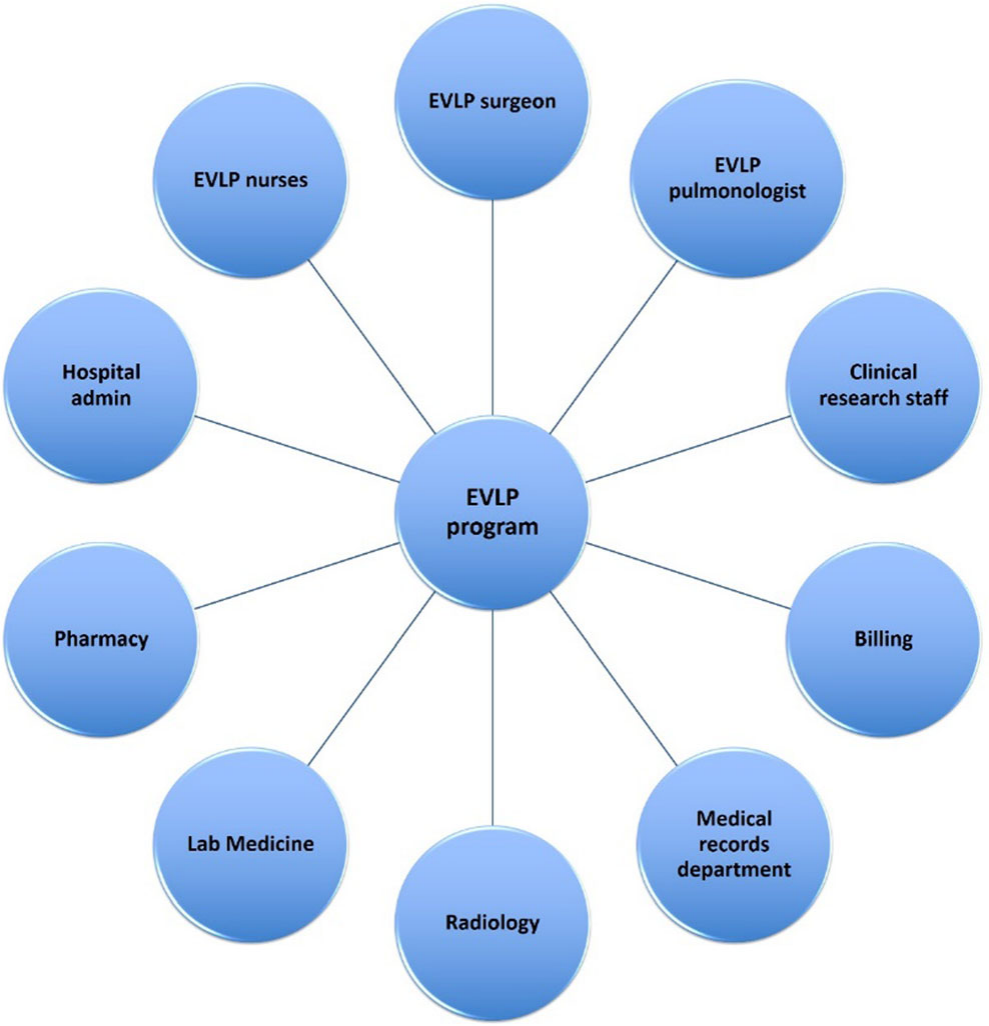
The multidisciplinary EVLP team (EVLP, ex vivo lung perfusion)

Given our current case volumes, we are unable to allocate full-time perfusionists for the EVLP program and sought out an alternative model. The guiding principle was to create a program aligned with the multidisciplinary structure of the lung transplant program (Fig. 2). The core leadership of the program included a lung transplant surgeon, pulmonologist, and nursing lead. The core group of nurses consisting of Cardiovascular Intensive Care Unit nurses (who are already familiar with extra corporeal membrane oxygenation (ECMO) and ventricular assist device (VAD) management) were trained to work as the lung perfusionists. The EVLP team was set up to be on call at all times. We were the first EVLP program in our state centered on this model.

In this review, we describe in detail the fundamentals of the EVLP program at our institution.

## Implementation of the program

Prior to initiating our EVLP program, we looked at existing programs to learn from their processes. We then acquired the XVIVO® perfusion system (XPS) machine (XVIVO Perfusion AB, Sweden), after which we began an extensive training process, which included orienting the neighboring organ procurement organizations, training nurses, radiology technicians, labs, surgeons, and pulmonologists. Additionally, 10 intensive care unit (ICU) nurses were trained to set up and run the machine in conjunction with our surgeons, pulmonologists, and research staff. As part of the program initiation, ten human lungs that had been declined for transplant were procured for research and training purposes. Specific protocols and training modules were designed to standardize the management of the machine. Following training runs, all personnel underwent a thorough credentialing process. We set up a roster of an EVLP surgeon, pulmonologist, and three EVLP nurses on call.

In summary, the steps were as follows:

1. Extensive meetings, involving all stakeholders, to develop protocols and procedures.
2. Meeting with the local organ procurement organization to educate, train, and obtain their buy-in to enable allocation of lungs via the EVLP route.
3. Visits to other sites performing EVLP.
4. Ten trial lungs (human lungs not considered for transplantation) were tested on the EVLP machine for training and establishing workflow, which was kept in line with the overall functioning of the transplant program.
5. Extensive training, including wet labs
6. Flashcards were developed with every minute step of the procedure, from the time of organ arrival to dispatching, in order to ensure reproducibility and minimizing errors.

### Skills assessment

All personnel who run the EVLP machine completed a skills assessment. Select surgeons also visited the University of Toronto and Indiana University for training. Similarly, several nurses went to other centers to receive additional training. After the completion of training, each individual had to be signed off on both his and her knowledge and skill.

### The workflow

**Fig. 3:**
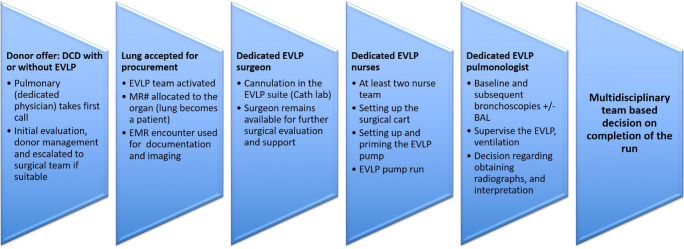
Typical workflow at our institution (MR#, medical record number; EMR, electronic medical record; BAL, bronchoalveolar lavage; DCD, donation after circulatory death; EVLP, ex vivo lung perfusion)

The workflow (Fig. 3) involves initial screening of the donor by the on-call transplant pulmonologist and the surgeon followed by the involvement of the EVLP pulmonologist and surgeon, if EVLP is being considered. Once lungs are accepted for EVLP, the on-call team is activated. The EVLP nursing team, per protocol, does the initial setup and subsequent running of the EVLP. After connecting the cannulas, the EVLP surgeon initiates the pump and remains available for gross assessment, pulmonary venous gas measurements if needed, and any adjustment to the cannulas or the endotracheal tube. The assessment and management of physiologic variables, as well as airway examinations, are the responsibility of the EVLP pulmonologist. The final decision to transplant is made in a collaborative manner by the EVLP team in consultation with the transplantation surgeon.

### Chain of command

- Organ procurement organization (OPO) informs the transplant surgeon. If DCD, EVLP team mobilized (on call 24/7). If donation after death, wait for input from procuring surgeon.
- Surgeon and often the pulmonologist travel for procurement to facilitate decision-making in the donor OR.
- If the lungs accepted for EVLP, the multidisciplinary team executes the EVLP run.
- The lung is allocated a medical record number (in other words, the lungs become a patient).
- We use a dedicated cardiac catheterization room for our EVLP run. The surgeon cannulates the lungs and connects to the EVLP machine. Available for surgical evaluation.
- Two on-call EVLP nurses run the pump and function as a scrub tech.
- A pulmonologist does the bronchoscopies and supervises ventilation, pump run, and radiographic studies.
- The lungs remain on the pump for at least 2 h and up to 6 h before a decision is made regarding proceeding with transplant or declining.

#### Preparation/setting up the XPS

**Fig. 4:**
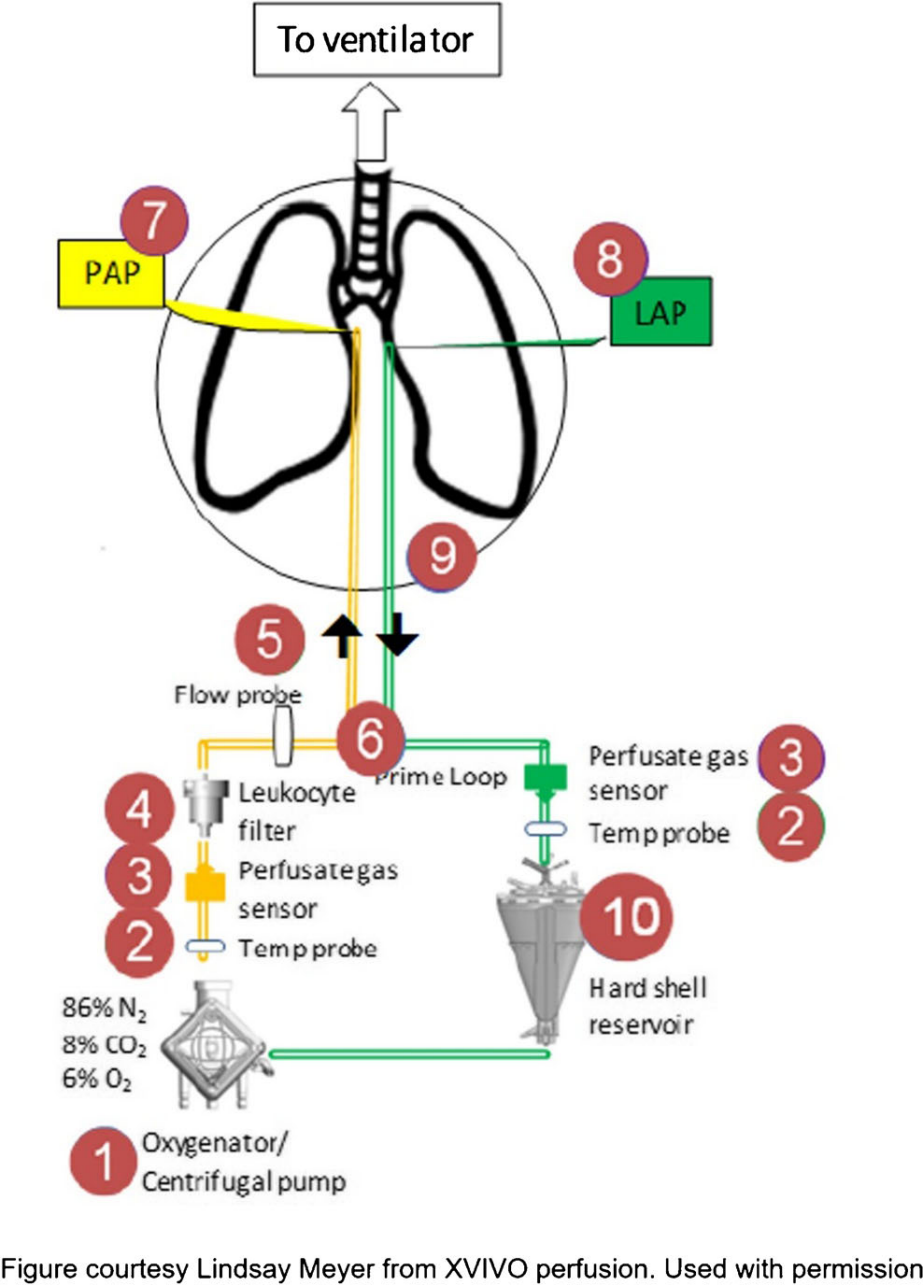
The EVLP circuit (EVLP, ex vivo lung perfusion; PAP, pulmonary artery prime; LAP, left atrial prime)

A typical EVLP circuit is shown in Fig. 4 (please refer to the product manual for more details (XVIVO Perfusion)). It consists of a centrifugal pump, which pumps the perfusate through the membrane oxygenator, leukocyte filter into the pulmonary artery (PA) (yellow tubing). A standard ventilator ventilates the lungs. The membrane oxygenator is connected to a gas tank, which consists a mixture of nitrogen (86%), CO_2_ (8%), and O_2_ (6%). The perfusate will move from the lungs to the reservoir via left atrial (LA) cannula and green tubing [20].

Prior to initiation of EVLP, it is essential to gather all necessary equipment and supplies. This includes at least eight bottles of Steen Solution (XVIVO Perfusion, Gothenburg, Sweden), 5 l of Perfadex solution (XVIVO Perfusion, Gothenburg, Sweden) in an ice cooler, drug box, iSTAT machine for blood gases, 15 cartridges, XVIVO disposable kit, slush machine, and a bronchoscopy cart. We remove three bottles of Steen Solution from the cooler and allow it to rise to room temperature. At this point, it is critical to maintain a sterile environment as you would in the OR (all personnel are to wear surgical hats, mask, along with sterile gowns and gloves). In addition to a surgeon, one of the EVLP nurses scrubs in to help with the cannulation.

**Fig. 5:**
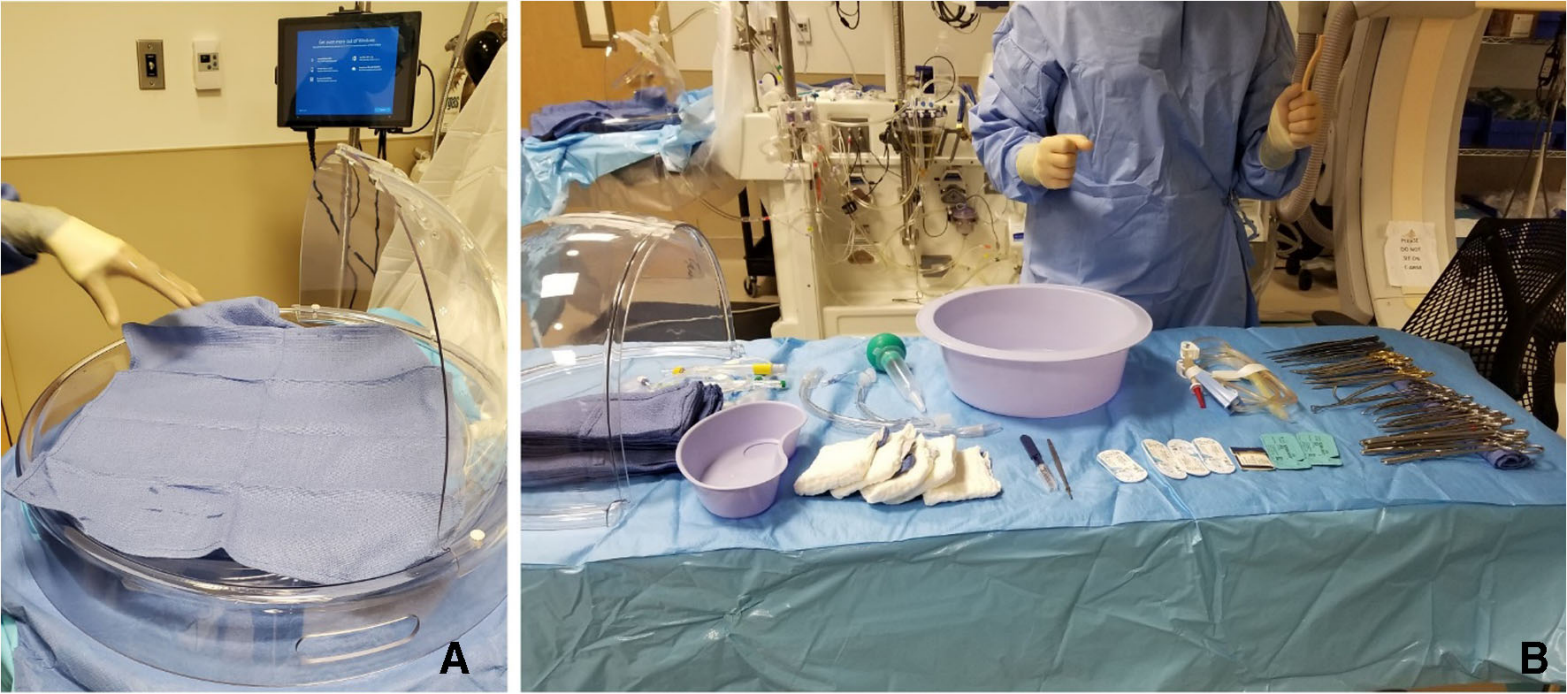
**A** The organ chamber is set up. **B** The back table with all instruments for cannulation

Preparation of the organ chamber requires a sterile nurse to place a U-drape on the organ chamber platform. The circulating nurse secures the drape with clamps from the XPS machine. Thereafter, the circulating nurse will open the outermost clear packaging of the organ chamber, remove it, and place it on the back table. The outer drape of the organ chamber is opened aseptically, forming the first layer of the back table. The organ chamber remains wrapped in its innermost layer until the surgeon arrives. During this time, a back table is set up with all instruments and supplies needed for the case (Fig. 5).

The XPS is turned on, and the perfusion circuit is set up. The ventilator is connected while ensuring that both the sweep gas and the oxygen are connected to the equipment. A standard heater/cooler is set up and set to 1 °F above the starting temperature. Three bottles of room temperature Steen Solution are added to the reservoir. The CardioHelp device is primed, and medications are added at this time (heparin: 10,000 units, meropenem: 500 mg, and Solumedrol: 500 mg). Finally, the ventilator is calibrated and at this point, the system is ready for organ arrival.

#### Organ preparation

After the arrival of the organ, a formal time-out is performed, and the United Network for Organ Sharing (UNOS) Label checklist is completed. The sterile nurse at this time unwraps the organ chamber while the surgeon removes the lungs from the organ bag leaving the innermost bag intact.

**Fig. 6:**
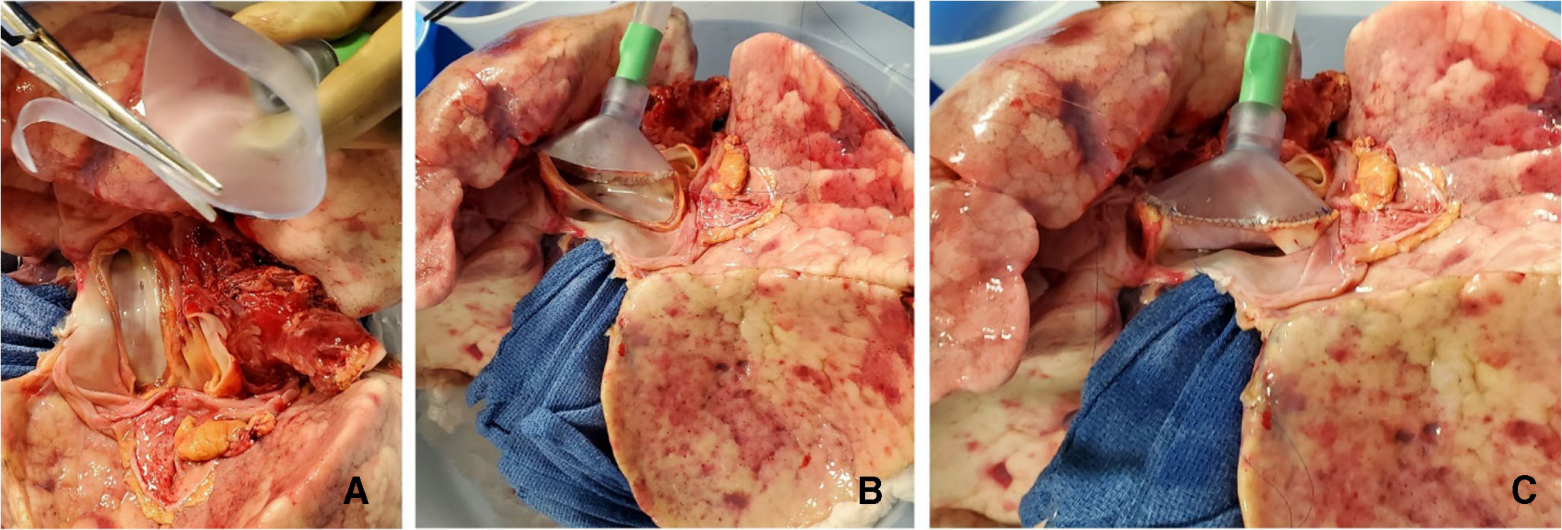
**A** XPS LA cannula trimmed to size. **B** XPS LA cannula sutured to LA cuff. **C** Finished anastomosis (XPS, XVIVO® perfusion system; LA, left atrial)

**Fig. 7:**
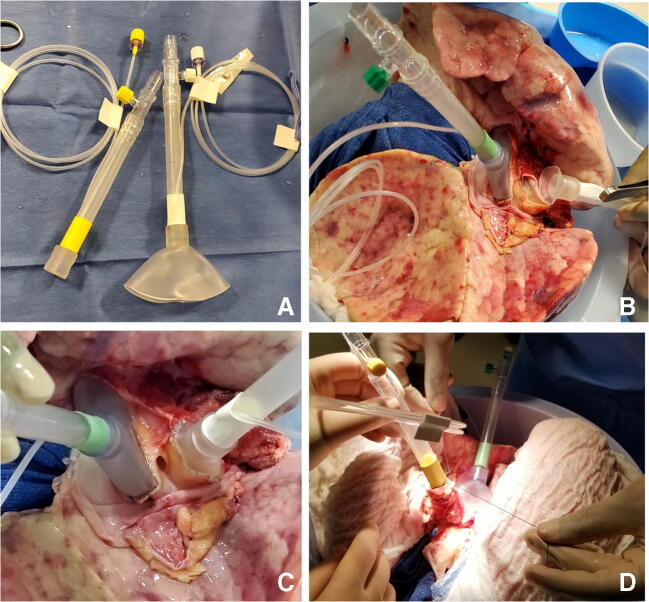
**A** Types of XPS PA cannulas. **B** XPS cannula sutured to PA cuff. **C** Completed LA and PA anastomoses. **D** If the native PA is recovered in the donor lung, then the yellow cannula is used to cannulate PA and secured with silk ties (XPS, XVIVO® perfusion system; PA, pulmonary artery; LA, left atrial)

**Fig. 8:**
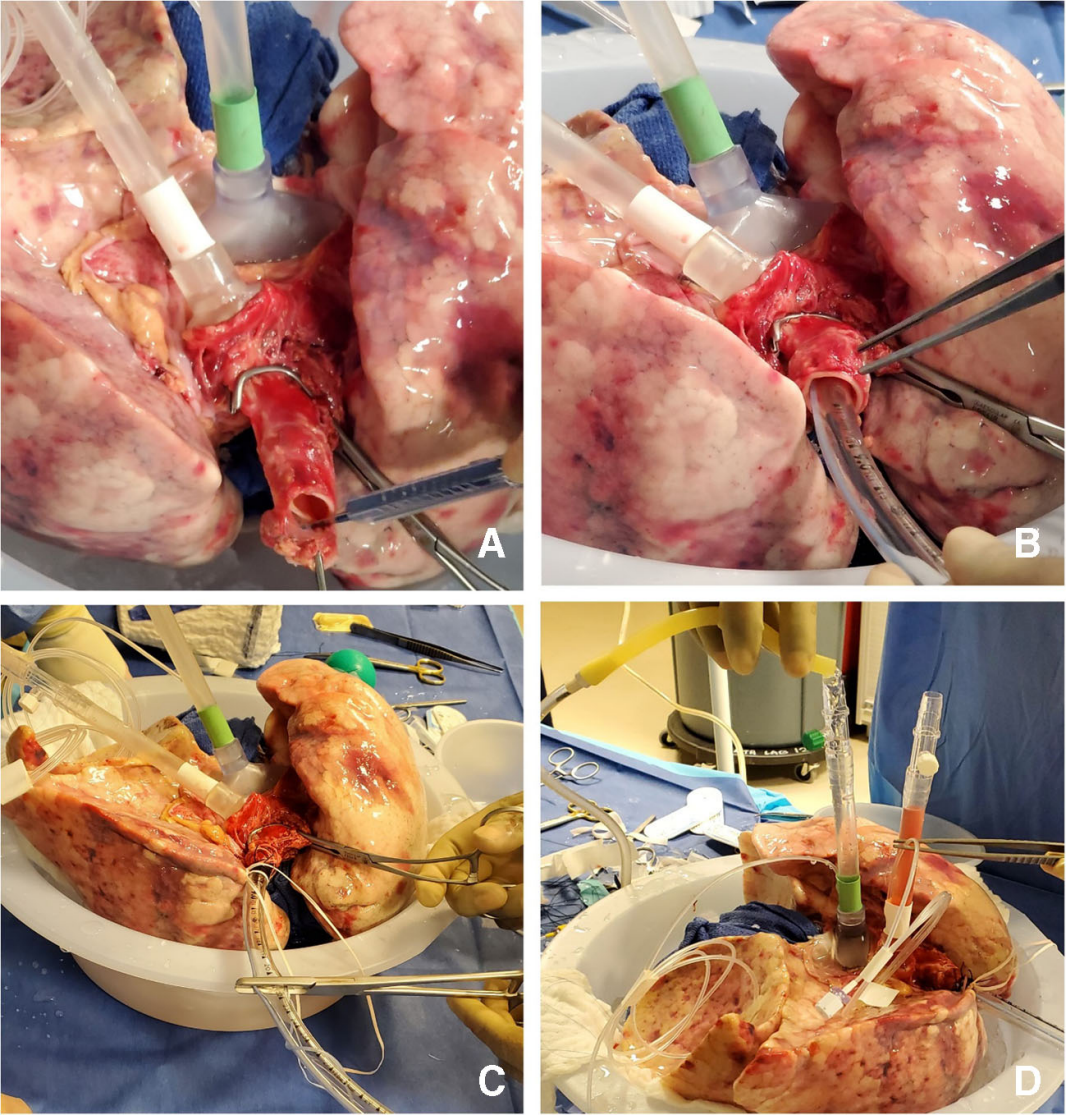
**A** Trachea is clamped with a Satinsky clamp and the stapled line removed. **B** Endotracheal tube inserted. **C** The ET tube is secured with silk ties and umbilical tape and ET tube clamped (remove the Satinsky clamp). **D** Retrograde flush with 1 l of Perfadex solution (ET, endotracheal)

Steps include the following:

1. The LA cuff is anastomosed to the XPS LA cannula (marked green) with a 5-0 polypropylene suture to ensure a watertight anastomosis (Fig. 6).
2. The PA cuff is sutured to the XPS PA cannula cuff (marked white). If the heart is not being recovered for transplantation, the entire main PA is procured with the lung en bloc, which facilitates insertion of the XPS cannula (yellow color) into the PA and tied over with silk sutures, obviating the need for suturing the cuff (Fig. 7).
3. The trachea is then clamped at the bifurcation with a Satinsky clamp and the staple line opened. A large (often no. 9 size) endotracheal (ET) tube with the cuff end cut is passed through the trachea and secured with three 0 silk ties and one umbilical tape. It is important to ensure that they are reasonably tight around the trachea and secured to the ET tube to prevent air leaks. The ET tube is now clamped as the Satinsky clamp is removed to ensure that the lungs are still maintained at the functional residual capacity (Fig. 8).
4. The ET tube remains clamped while a retrograde flush is performed with 1 l of cold buffered Perfadex infused into the LA cannula (Fig. 8D). This allows for inspection of the anastomosis for any leaks. Once the flush is completed, the lungs are transported to the XPS sterile basin.

#### Initiating retrograde flow

**Fig. 9:**
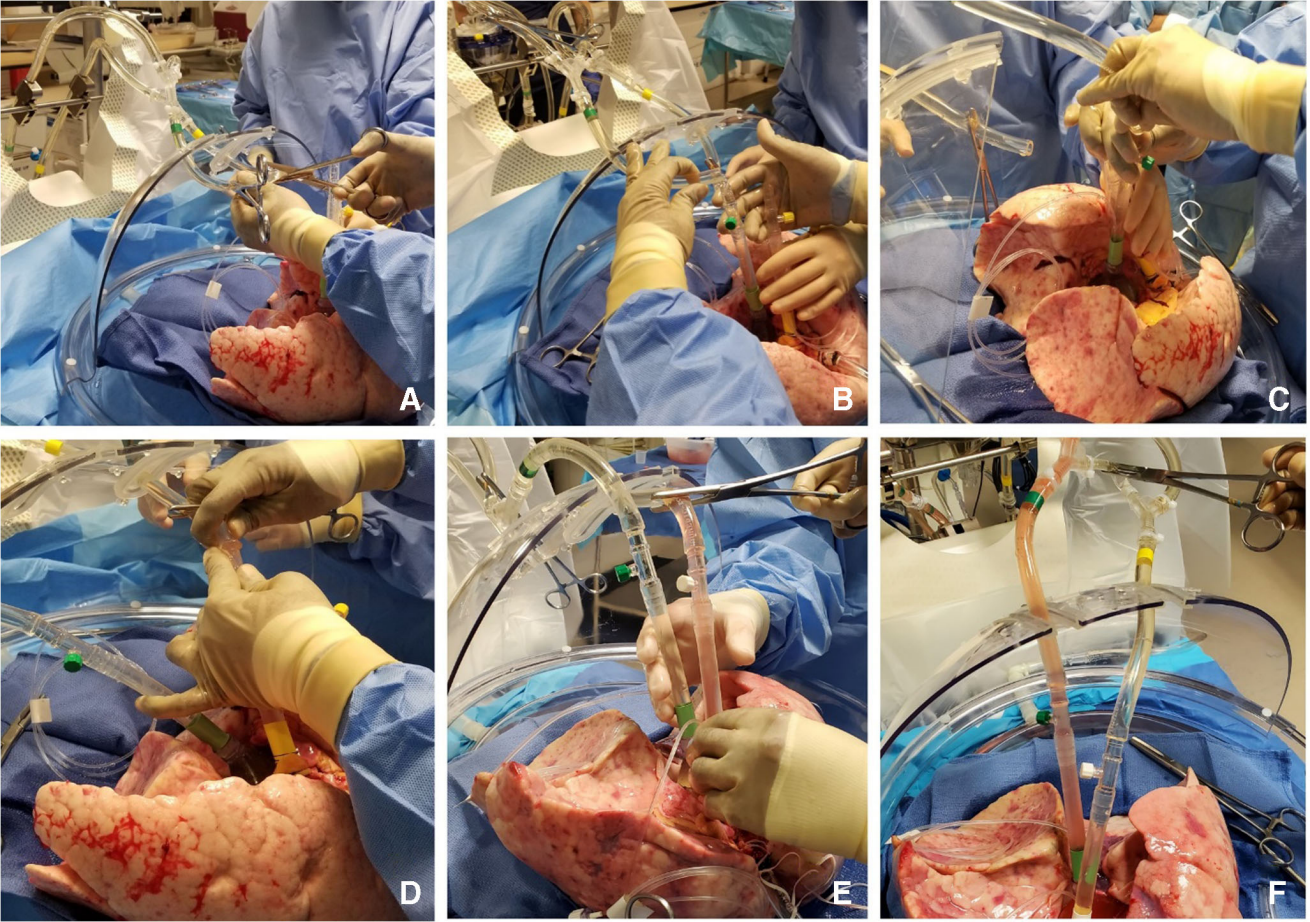
**A** The circuit divided between clamps. **B** LA line (green) proximal to the bridge is clamped and second clamp on distal PA tubing (yellow). Retrograde flush from LA to PA done. **C** LA cannula connected. **D** PA tubing de-aired and PA cannula connected. **E** PA cannula removed. **F** LA clamp removed and placed on the bridge thus initiating the flow (LA, left atrial; PA, pulmonary artery)

The sequence of initiating flow is shown in (Fig. 9). The sequence involves first dividing the circuit between clamps, then placing a clamp on the LA line proximal to the bridge and a second clamp on the distal PA tubing. This redirects the flow to go in the LA cannula and out the PA cannula. The LA cannula is flushed while the tubing is connected. The CardioHelp pump is turned on at 650–700 revolutions per minute (RPMs) and increased slowly to fill the LA cannula with Steen Solution while taking care not to entrain air. The Steen Solution is flushed through the lungs in a retrograde fashion until the solution runs clear and is free of clots and debris.

While continuing to flush the Steen Solution, the PA cannula is de-aired and connected to the PA tubing. The flows are decreased and the clamps are removed from the LA and PA lines and the bridge is clamped, which initiates flow through the lungs.

#### Initiating antegrade flow

All transducers are leveled and zeroed. The CardioHelp pump is adjusted to achieve flows according to the EVLP protocol for the first 10-min interval. During this time, changes are made to the flow and temperature according to the protocol, until the target temperature and flows have been achieved.

#### Ventilation

**Fig. 10:**
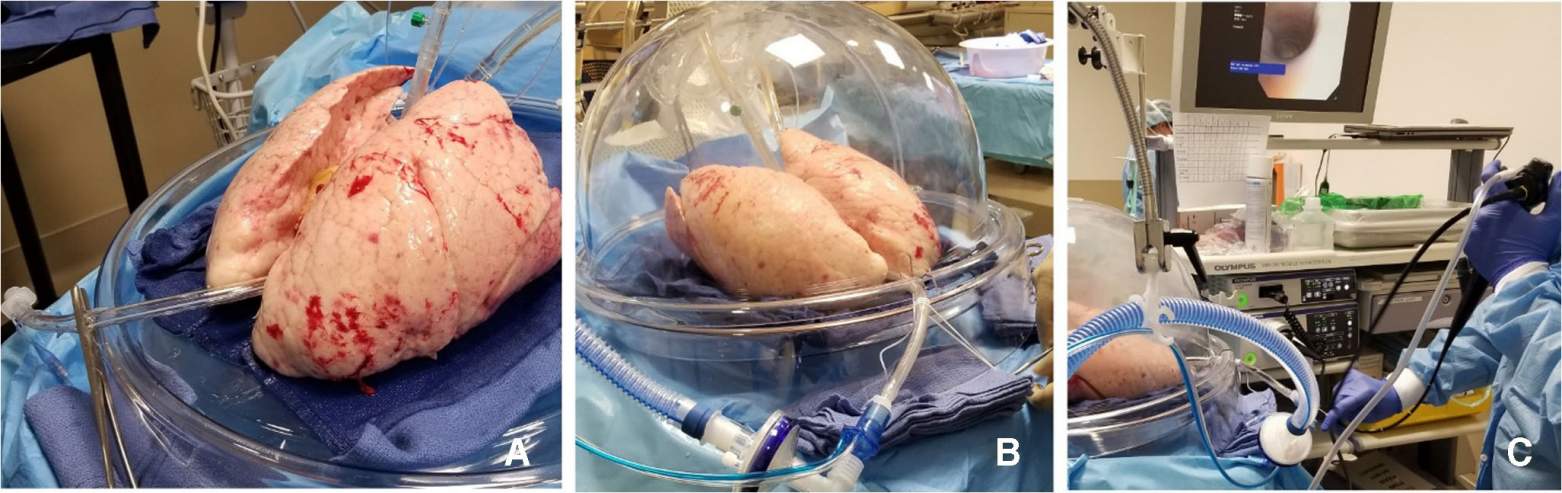
**A** ET tube is clamped during rewarming. **B** Ventilation started when the LA temperature reaches 32 °F. **C** Bronchoscopy being performed

Once the temperature of the LA effluent reaches 32 °F, ventilation is initiated (Fig. 10).

We follow the routine protocol described in literature for management of EVLP pump [20]. Recruitment maneuvers with 100% fraction of inspired oxygen (FiO_2_) challenge are performed at 50 min into the protocol and every hour thereafter. Bronchoscopy can be performed at any point, typically prior to recruitment, at the discretion of the EVLP pulmonologist. During the recruitment maneuvers with O_2_ challenge, the FiO_2_ is increased to 1.0, respiratory rate is increased from 7 to 10 breaths per minute, and the tidal volume is increased from 7 to 10 ml/kg (based on the ideal body weight of the donor). During the last minute of the recruitment maneuver, a perfusate sample is drawn from the LA and PA cannulas for analysis. A baseline chest X-ray is performed at this time.

Additional recruitment maneuvers are performed by maintaining the airway pressure to 25 cm H_2_O for 15-s intervals at the discretion of the EVLP pulmonologist.

#### Monitoring and maintenance

Steen Solution is added to the reservoir as needed with additional doses of heparin, meropenem, and Solumedrol. Typically, one fresh bottle of Steen Solution is added each hour while draining a similar amount from the circulating perfusate. The ventilation and perfusion are continued per the EVLP protocol. An LA pressure of 3–5 mmHg is maintained. Recruitment maneuvers are performed at the end of every hour, and perfusate samples are drawn at the conclusion. Bronchoscopy is repeated as desired, and a chest radiograph is repeated prior to the conclusion of EVLP to assess the impact on parenchymal opacities.

### Assessing transplant suitability

#### Post-EVLP transplant inclusion criteria

**Fig. 11:**
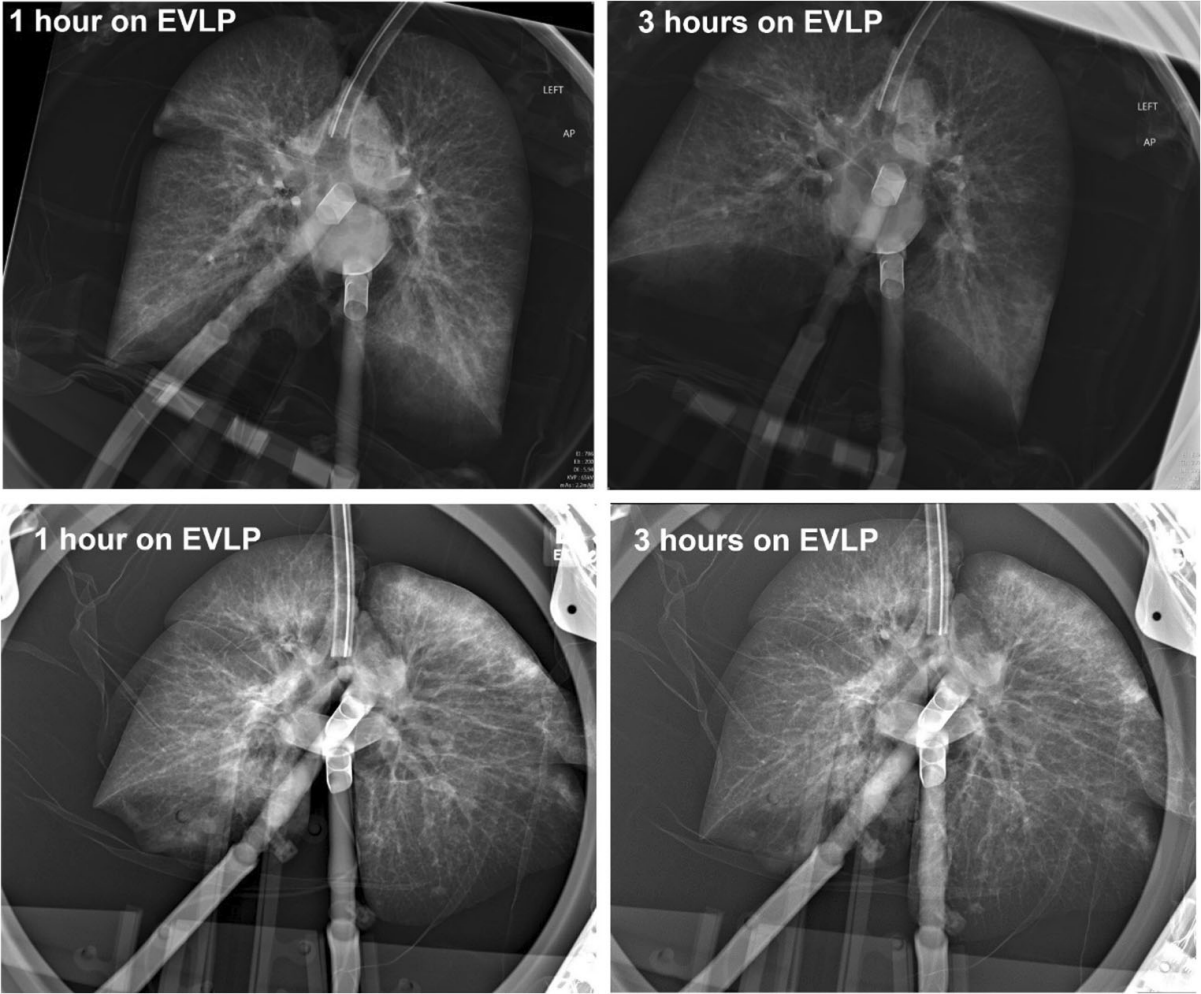
CXR of a successful EVLP run (CXR, chest X-ray; EVLP, ex vivo lung perfusion)

- The surgeon’s evaluation of the lungs includes overall improvement in function and a test of elastance, when the ventilator is disconnected. Good compliance, ballotability of lungs, no visible consolidation, and increased “bogginess” are other criteria used to determine the quality and transplantability of the lungs.
- The pulmonologist evaluates the airway and checks for evidence of Steen Solution leak into the airways. Stability or improvement of other pulmonary physiologic parameters during EVLP, including the pulmonary vascular resistance (PVR), dynamic and static compliance, and airway pressures.
- Two ΔPa O_2_ (LA Pa O_2_ – PA Pa O_2_) ≥350 mmHg
- If two delta ΔPO_2_ ≥ 350 mmHg are not met, three out of the following four parameters must be present:
  i. One ΔP O_2_ > 350 mmHg or absolute LA PO_2_ > 400 mmHg.
  ii. Chest X-ray findings with the absence of or improvement of pulmonary edema/infiltrates (Fig. 11).
  iii. Compliance-static (>35 for a single and > 60 for a double).
  iv. Absence of consolidation by palpation.

#### Post-EVLP transplant exclusion criteria (Fig. 12)

- All ΔP O_2_ < 350 mmHg (measured with FiO_2_ set at 1.0) or absolute LA PO_2_ < 400 mmHg.
- Overall greater than 10–15% functional deterioration of all the pulmonary physiologic parameters (PVR, compliance, airway pressure) with chest X-ray findings indicating deterioration.
- Steen leak from airway
- Increased bogginess or edema of the lungs

**Fig. 12:**
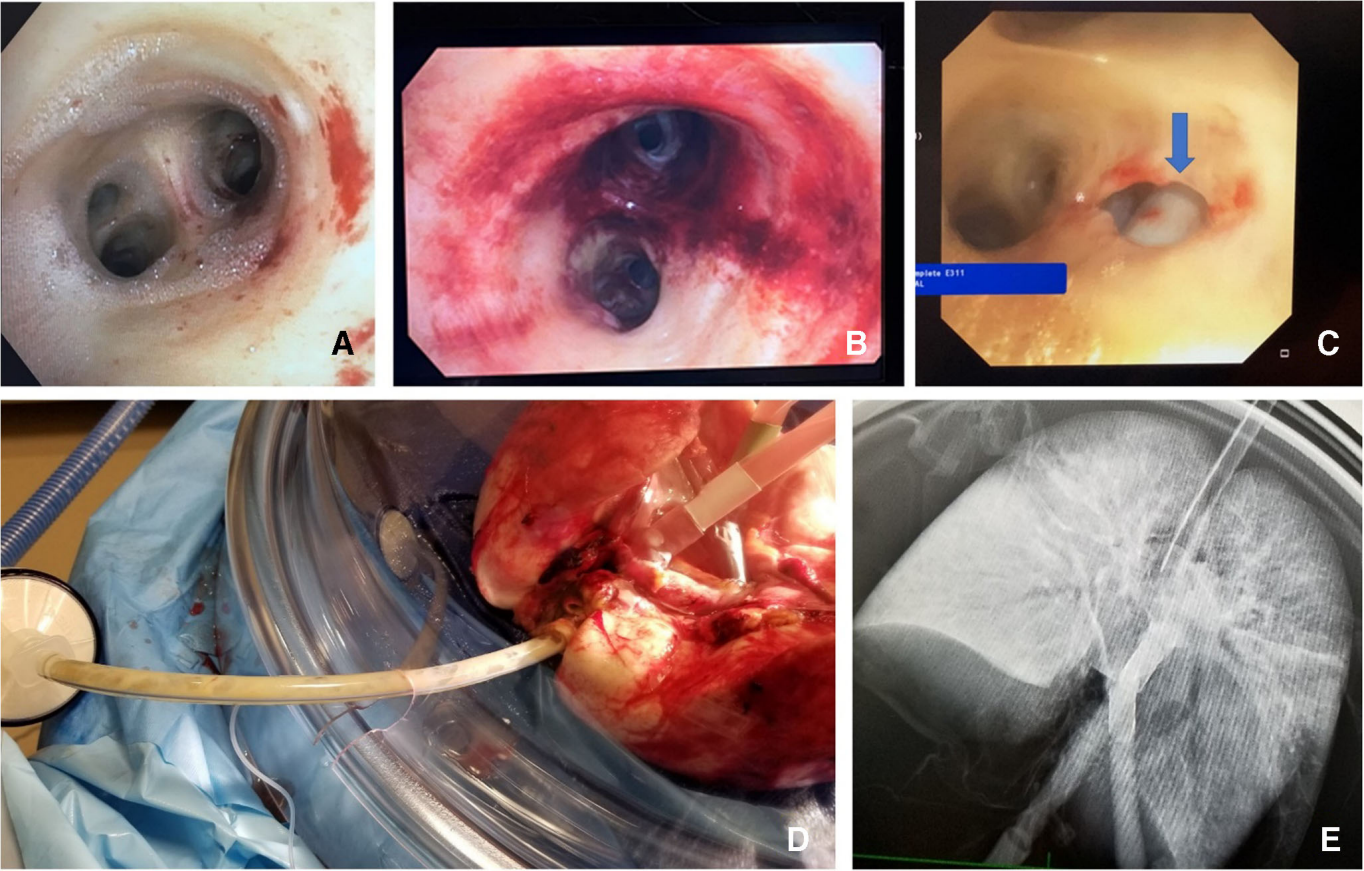
**A** Steen leak seen on bronchoscopy. **B** Extensive airway damage. **C** A tear in the bronchus in a DCD patient, which was missed on initial bronchoscopy but detected on EVLP assessments. **D** Progressive bogginess in the right lung with Steen and purulent secretions through ET tube. **E** CXR of the same lungs showing extensive consolidation in the right lung (EVLP, ex vivo lung perfusion; DCD, donation after circulatory death; ET, endotracheal)

##### Repackaging the lungs

**Fig. 13:**
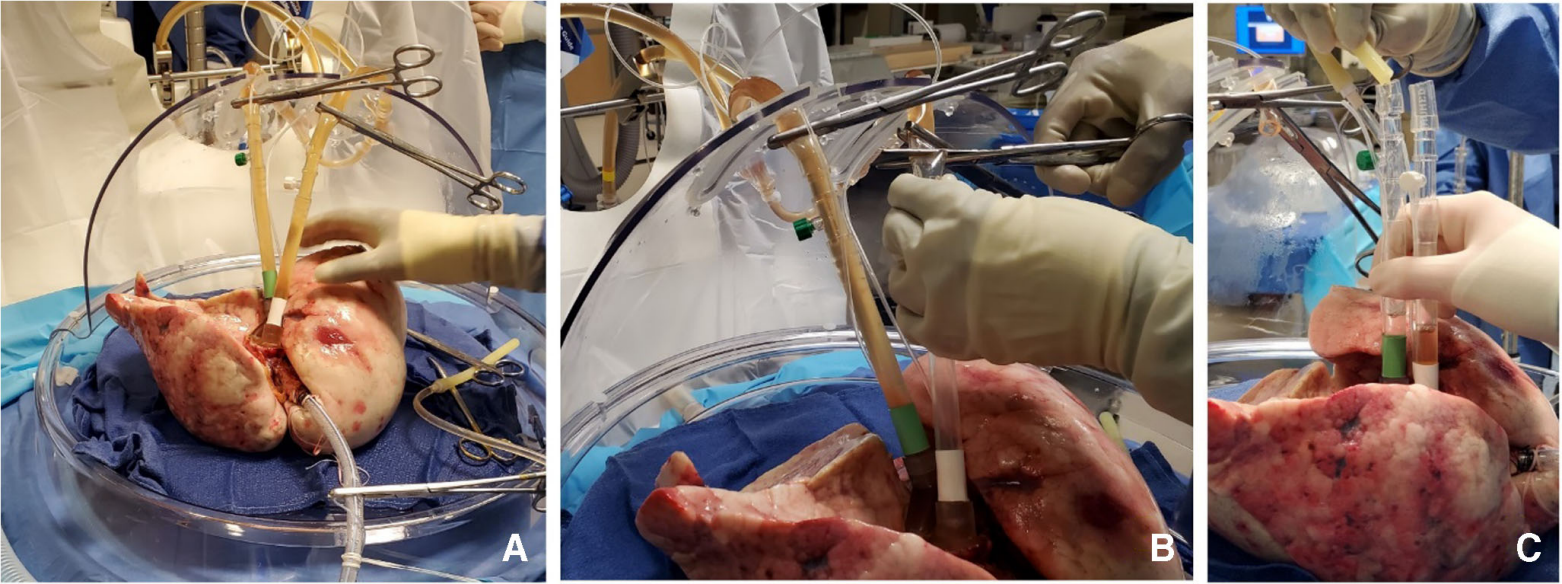
Sequence of decannulation. **A** Both LA and PA lines clamped, ET tube clamped. **B** Cannulas disconnected. **C** Retrograde perfusion started (LA, left atrial; PA, pulmonary artery; ET, endotracheal)

The heater/cooler is turned to 15 °F and the FiO_2_ is decreased to 0.5. Once the lungs reach 32 °F, the cannulas are clamped and disconnected from the circuit (Fig. 13).

**Fig. 14:**
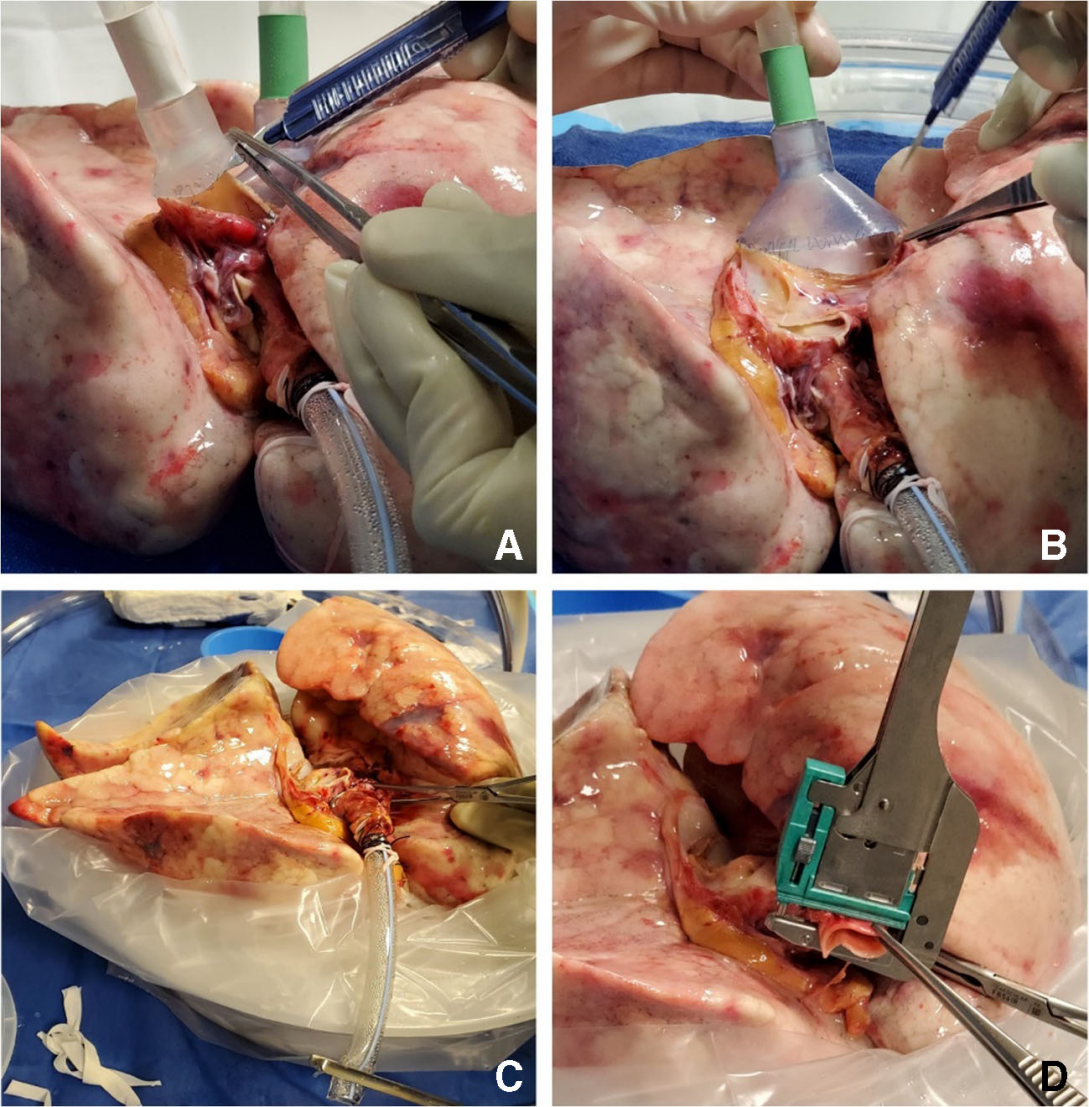
**A** PA cannula disconnected. **B** LA cannula disconnected. **C** Satinsky clamp of trachea and ET tube removed. **D** Trachea stapled with TX-30 green stapler (Ethicon PROXIMATE TX Reloadable Linear Stapler) (PA, pulmonary artery; LA, left atrial; ET, endotracheal)

Two breaths are given; the ET tube is clamped and disconnected from the ventilator. A retrograde perfusion is done with 1 l of Perfadex solution and the cannulas are removed. The trachea is clamped with a Satinsky clamp, the ET tube is removed, and the trachea is stapled with a TX-30 green stapler (Ethicon PROXIMATE TX Reloadable Linear Stapler) (Fig. 14). The lungs are then repackaged in Perfadex solution.

**Fig. 15:**
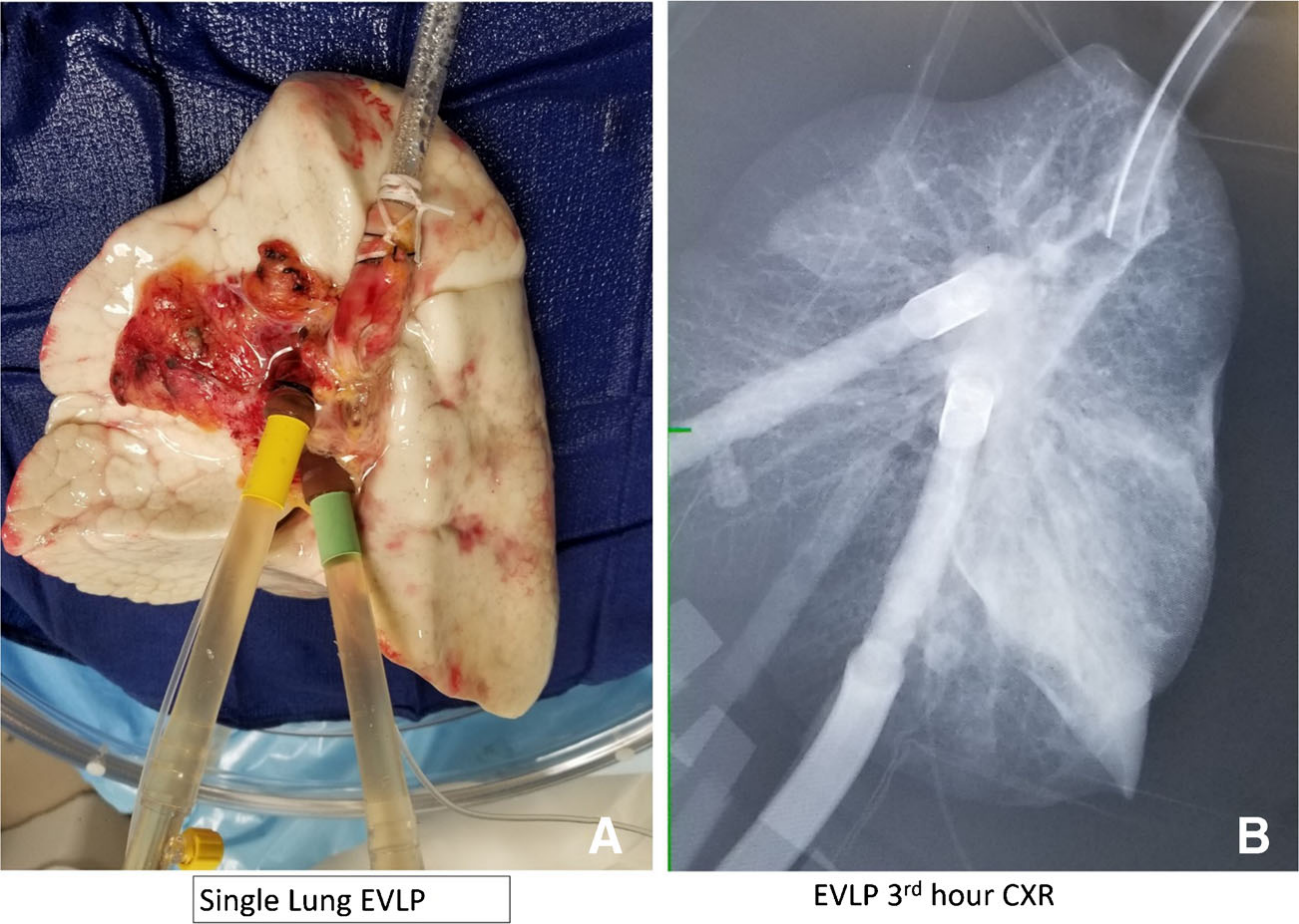
**A** Single lung EVLP run. **B** CXR after 3 h showing dense consolidation in the lower lobe (EVLP, ex vivo lung perfusion; CXR, chest X-ray)

If the intention is to perform single lung transplantation, we generally treat both lungs with EVLP. However, if the other lung is unrepairable, single lung EVLP can be done. We adjust all the settings for single lung flows. Figure 15 shows one such case in which the lung was not suitable for transplantation at the end of 3 h.

## Our experience

After the practice runs with rejected human lungs and personnel credentialing, the program was cleared to start clinical cases in early 2016. The DCD program was also started at the same time.

In April 2016, our center became the first center in our state to conduct the first lung transplant reconditioned using the EVLP technology. This was also the first DCD donor utilized for lung transplantation at our institution. The recipient had no evidence of primary graft dysfunction and continues to do well to date.

At the time our program started, the EVLP technology was yet to receive full Food and Drug Administration (FDA) approval for regular clinical use and was being utilized under the humanitarian device exemption status. The EVLP program at our university was approved to start as one of the centers for NOVEL clinical trial and had to follow the study protocol for donor lung utilization. Since the lungs were technically being used for “research”, lungs were offered for EVLP only after they had been turned down by all the centers for clinical use. This created a significant limitation in the program’s ability to utilize the technology, as most of the offered lungs were unsuitable for EVLP. This changed in April 2019, when the XPS device received the FDA market approval, which permitted the allocation of donor lungs for EVLP technology to proceed routinely. The temporal trends in the number of lungs considered and eventually placed on EVLP for reconditioning are reflective of these developments.

The early experience at our institution was one of “cautious optimism”. To date, we have activated EVLP 58 times. However, only 24 lungs were placed on EVLP, as many lungs were deemed too poor to be placed on EVLP or some DCD patients did not die within allocated time. Of the 24 EVLP runs, 17 lungs were transplanted. This is an acceptance rate of 71% from EVLP. During this entire period, we performed 343 lung transplants and so the overall EVLP rate is about 5%. It is beyond the scope of this paper to discuss the long-term outcomes. Of the 17 transplants, we had one in-hospital (30-day) mortality due to massive cardiac embolic stroke. The 6-month survival of the rest has been 93.75%.

The recipients transplanted using EVLP technology have included high-risk recipients, such as those bridged to lung transplant on mechanical ventilation and ECMO support. The recipient profile and the early outcomes among patients transplanted using EVLP are similar to other patients transplanted without EVLP during the same period. Therefore, EVLP technology has been a valuable addition to our program as it has facilitated a significant proportion of incremental transplants (5.2% overall, 12.5% since 2019, when EVLP obtained FDA approval).

Some challenges for our EVLP program included the high attrition rate on the nursing side with a need for continual training and credentialing of the new personnel. To date, we have regular monthly wet labs to maintain skills for our nurses. Additionally, the technology is expensive, with significant fixed and variable costs. Despite the novelty and the cutting-edge nature of the technology, the cost aspect becomes an important concern, especially when the equipment is used infrequently. Finally, despite all the protocols, training, and practice runs, the EVLP technology involves a steep learning curve. The EVLP runs rarely tend to be smooth and uneventful, requiring close monitoring and the presence of the EVLP physicians in the EVLP suite.

## Conclusion

The EVLP technology is a promising tool to tackle the challenge of high waitlist mortality among patients awaiting a lung transplant. EVLP technology brings about a paradigm shift in the process of organ preservation by allowing assessment and reconditioning of the donor lungs in an ex vivo environment. As discussed extensively in the literature as well as in our own experience, the value of EVLP in expanding the donor pool is beyond the actual cases we pump. We are now able to consider and accept more extended criteria organs for evaluation with the knowledge that EVLP is available, in case it is needed. We may not use the EVLP in a substantial proportion of these organs, but in the absence of EVLP, we may be reluctant to travel. Our absolute EVLP activation numbers as well as the ratio of EVLP activation to EVLP utilization are reflective of this trend.

Despite early challenges related to a combination of regulatory restrictions, unfavorable donor allocation processes, and staff attrition, the EVLP program at our center has contributed to program growth by increasing lungs available to transplant at our program. Apart from allowing diagnostic and therapeutic interventions among donor lungs, EVLP holds great promise as a platform for future research in the field of pulmonary medicine. However, no innovation is complete without universal adaptation. Now this technology is resource intense and cost prohibitive in many centers across the world. Making this technology simple and affordable would make it more universally acceptable.
